# How age and health status impact attitudes towards aging and technologies in care: a quantitative analysis

**DOI:** 10.1186/s12877-023-04616-4

**Published:** 2024-01-03

**Authors:** Julia Offermann, Wiktoria Wilkowska, Thea Laurentius, L. Cornelius Bollheimer, Martina Ziefle

**Affiliations:** 1 Chair for Communication Science & Human-Computer Interaction Center, RWTH Aachen University, Campus-Boulevard 57, 52074 Aachen, Germany; 2 Chair of Geriatrics & Department of Geriatric Medicine, RWTH Aachen University Hospital, Aachen, Germany

**Keywords:** Technology acceptance, Aging, Care, Assistive technology, End-of-life decisions, Quantitative analyses

## Abstract

**Background:**

Increasing proportions of geriatric patients pose tremendous challenges for our society. Developments in assistive technologies have the potential to support older and frail people in aging and care. To reach a sustainable adoption of these technologies, the perceptions and wishes of future users must be understood. In particular, the relationships between individual health-related factors, and the perceptions of aging and using assistive technologies in severe health situations must be empirically examined.

**Methods:**

Addressing this research gap, our quantitative study (*N* = 570) investigates the impact of diverse future users’ age and health status on their a) perceptions of aging, b) perceptions and acceptance of using assistive technologies in aging and care, as well as c) end-of-life decisions regarding technology usage. For this, four groups were segmented for the comparison of younger (< 50 years) healthy, younger chronically ill, older (50 + years) healthy, and older chronically ill participants.

**Results:**

The results revealed that health status is more decisive for age-related perceptions compared to age. The technology-related perceptions were slightly impacted by either chronological age or health status. The end-of-life decisions showed the most striking differences in the willingness to use assistive technologies, revealing older chronically ill participants to have more restrained attitudes towards technology usage than older healthy as well as all younger participants.

**Conclusions:**

The findings suggest that the benefits of assistive technologies in private or professional care contexts should be communicated and implemented tailored to the respective user group’s needs. Moreover, the results allow us to derive practical implications within the geriatric care context.

**Supplementary Information:**

The online version contains supplementary material available at 10.1186/s12877-023-04616-4.

## Introduction

The course of demographic change has led to increasing proportions of older people in need of care. This development poses high burdens for the care sector due to a growing lack of individuals being formally or informally able to care for all those needing care [1–3]. Therefore, it is required to develop solutions that aim at the support of older people in their everyday lives, maintening of being active and independent, and relieving their caring persons in private as well as professional care environments. One approach refers to the development of assistive technologies that support older people using monitoring their health changes and staying within their loved home environments [4–7]. In this regard, diverse ambient assistive technologies and systems are designed, reaching from specific technologies to holistic systems and platforms [8]. Beyond these technical opportunities, the users’ acceptance is essential for the sustainable adoption and usage of these technologies. Research has therefore increasingly focused on future users’ acceptance and perception of assistive health-related technologies [9, 10]. Besides individual characteristics, such as chronological age and health status, that significantly influence the willingness to adopt medical assistive technologies [9, 11], research revealed that aging-related perceptions are also decisive for health behavior and related attitudes [12]. Considering these insights, it is not known so far to what extent the chronological age and health as connected user factors impact a) aging-related perceptions, b) perceptions of using assistive technology in the aging and care context, and c) decisions about technology usage in severe health (end-of-life) situations. Therefore, the current study investigates these potential relationships within an online survey approach, applying a four-field scheme that refers to the participants’ age and health status, differentiating between younger healthy, younger chronically ill, older healthy, and older chronically ill participants. This scheme was applied to investigate the connected impact of age and health status on the perceptions of aging as well as the evaluation and acceptance of using assistive technologies in older age. Within the next subsections, the theoretical background on aging and the acceptance of assistive technology is presented from the medical and social science perspectives. Afterward, the underlying research questions are derived based on previous research in the field. Finally, the research gap and the aim of this study are summarized.

### Medical and social perspectives on aging

Aging denotes a cumulative process due to permanent anabolic and concurrent katabolic events during life [13]. The term biological age depicts a snapshot at a certain point of chronological age. That functional state results from salutogenetic resources, capabilities, and resilience [14] but also from impairments, disabilities, and handicaps. The latter coins the term biological age with a rather negative connotation: Although biological age is an indisputable function of advancing chronological age, it is afflicted by an impenetrable maze of intervening variables which makes the life phase of aging interindividually so heterogenous [15]. Against this background, geriatrics caring for biological, psychological, and social health is guided not too much by the chronological but instead primarily by the biological age. The geriatric assessment is used to detect biological age. As part of the assessment, various domains such as mobility, memory, mood, and self-help ability are examined and standardized. As patients have an identical calendar age but therefore a different biological age, this is also referred to as the heterogeneity of the major life phase of ageing [16].

Geriatric patients are within their grand life phase of aging, which lumps together the subgroups of young-olds (65–74 years) middle-olds (75–84 years), and old-olds (85 + years) [17]. In the middle-olds and old-olds the corresponding biological age often appears adequate—or at least somehow expected [18]. Conversely, in young-olds, and already at the brink of being old (50 +), biological age rather occurs as an approaching issue, which has to be countered by preventive or compensating measures such as assistive technologies. The immediacy of this threat might be aggravated by an additional and gradual occurence of chronic disease(s) and thereby cause different reflections about the situation of a full-blown loss of autonomy or in terms of end-of-life decisions. Compared to younger patients, geriatric patients also frequently exhibit multimorbidity, which is characterized by the presence of several illnesses. Very old patients are also characterized by increased vulnerability to acute illnesses with a risk of deterioration in self-help and functional status [19, 20].

Accompanying the medical perspective on aging and besides the chronological age of people, the social perception and consideration of aging in terms of people’s perceptions and (positive and negative) attitudes towards aging received increasing attention in recent years [12, 21, 22]. Indeed, aging is a highly complex and individually diverging process that is frequently connected to negative aspects, such as a decrease in physical, cognitive, and mental skills, leading to high frailty and rendering persons affected vulnerable to increased risk of hospitalization, dependency in activities of daily living, social isolation, and institutionalization (e.g. [23–25],). In particular, negative perceptions of aging have been proven to be relevant predictors of decline in physical and psychological health in older age [22]. On the other side, aging is also increasingly associated with optimism, higher interpersonal trust, and well-being [26] as well as with growing perceptions of chances to age autonomously in active and healthy living [27, 28]. Thus, sociologically speaking, the concept of one’s own aging broadly differs among (older) individuals, and the perceptions of healthy and fortunate aging should be considered a multidimensional construct [29]. Studies revealed that the health status and existing physical impairments can considerably impact aging-related perceptions [30]. Research has shown that the different positive and negative attitudes towards aging can be significantly impacted by the participants’ age [21, 31], and have the potential to modulate the openness to use medical technology [32]. In addition, diverse approaches focused on vitality in older age [33–35] as a central parameter of wellbeing, or investigated vitality in the context of medical health surveys [36].

Beyond these insights, there is hardly any knowledge about the connected influence of an individual’s age and health status on aging-related perceptions. Therefore, the first research question that resulted in this study intended to reveal the following:

- RQ1: does the perception of aging-related aspects (i.e., perceived vitality, positive and negative effects of aging) differ depending on the participant’s age and health status?

### Developments in assistive technology and how to measure their acceptance

Over the last few decades, ambient and assistive technologies have been developed to support older persons and individuals needing care to enable aging within their home environment, remain as independent as possible, and stay socially and physically active [4, 37]. Some examples of assistive technologies refer to alarm and monitoring applications aiming for enhanced safety by the detection of falls and emergencies, typically using two components, such as a wearable device and a mobile phone (e.g., [38]). However, these applications can be used in a multitude of different forms ranging from (wearable) sensors [39] over video-based systems [40] to sensor-based systems [41]. Besides functions of facilitating communication with family, friends, and medical personnel [42] as well as memory functions [43], assistive technologies also aim at health monitoring to detect changes concerning the health status itself, movement patterns, and sleep rhythm [44, 45].

Beyond that, those applications are primarily developed to support and assist older adults staying longer within their own home environment (e.g., [4, 41, 43]), whereas numerous of these applications can also be applied in professional care settings, such as hospitals or nursing homes (e.g., [4, 37, 42]), to support professional caregivers and patients in their everyday life. Hence, the application potential and contexts are extremely broad and promising [4, 37].

Since there is currently a rapid development of health-related technology innovations, including ambient assisted living, lifelogging technologies, and gerontechnology, the sustainable adoption of such assistive technologies in information technology (IT) has been a major concern for research and practice. Many theoretical models have been proposed to examine the adoption and to predict the use of these technology innovations as reliably as possible. The most frequently used models in this context are the Technology Acceptance Model (TAM; [46, 47]) and the Unified Theory of Acceptance and Use of Technology (UTAUT; [48, 49]). Over the years, these models were adapted, extended, applied, and validated in diverse contexts and considering different user groups [10]. They represent the theoretical framework, providing factors that can explain acceptance and the use of the technologies to a great extent. TAM has become the dominant model for investigating factors affecting users’ acceptance of novel technical systems and presumes a mediating role of the perceived ease of use and perceived usefulness in the association between system characteristics (external variables) and system usage [10]. UTAUT is based on an analysis of existing technology acceptance models, such as TAM, TAM2, Diffusion of Innovation Theory, and Theory of Reasoned Action, aiming at consolidating these into one unified model that can assess the likelihood of success for new technologies and understand drivers of acceptance [50]. Both models focused on better understanding of why users accept or reject the different technologies and predicting their usage. However, even though these models provide a comprehensive theoretical framework, in recent years these technology acceptance models also received criticism for disregarding possible fluctuations over time [9]. The main weak point bears upon the fact that the models have been neither developed within a healthcare setting nor adjusted to this topic. Studies on TAM based on the research of IT and word processing systems [47], while UTAUT used studies related to an online meeting manager, a database application, and an accounting system [48]. Thus, these types of applications are not directly comparable to much more complex health-related technologies referring to computerized physician order entry, electronic health records, or nursing documentation systems [50]. Therefore, a more explorative approach is necessary to address the current requirements and wishes of users in the sensible context of aging and care.

In recent years, numerous studies have investigated the acceptance of health-related assistive technologies [9, 51, 52]. Diverse studies identified relevant motives and barriers of technology usage [5, 53, 54] and quantified technology-related perceptions and acceptance [10, 55]. In the decision to use or not use medical technology in the end, a recent study investigated the balancing of motives and barriers to technology usage for different person involved in the care process [56]. It is also relevant to analyze individual characteristics of users as potential influencing parameters on technology acceptance and perception. Here, research has focused on older users to do justice to their specific needs and requirements with regard to using technologies as a support for aging in place [5, 57, 58]. Further, previous research identified occasional effects of the participant’s health status on evaluations, perceptions, or acceptance of health-related technologies [59, 60]. Beyond these relationships, a direct interaction between age and health status – in terms of a group comparison, e.g., in a four-field scheme – has been disregarded so far and could deliver interesting insights related to the acceptance of using health-related assistive technology in older age. Based on this research gap, the second research question was derived:

- RQ2: do perceptions and acceptance of using assistive technology in old age differ depending on the participants’ age and health status?

### Connecting aging and technology acceptance by investigating end-of-life decisions

In later phases of life, aging and the need for support (e.g., using assistive technologies) are merging increasingly.

Health-supporting assistive technologies can fulfill particular medical safety-relevant functions, such as the detection of emergencies and monitoring of different vital parameters for individuals suffering from severe health conditions that can be critical to their lives, e.g., chronic heart failure patients [61], persons with chronic obstructive pulmonary disease [62], or individuals living with a kidney failure [63].

The moral, ethical, and other individual questions of what technology may and may not do in very late phases of life or end-of-life situations are becoming increasingly important. Previous research in this context focused on retrospective analyses investigating reasons for the death of patients following proportions of patients who decided to end their lives [64, 65]. Other studies examined people’s attitudes towards life-prolonging care [66, 67] and revealed significant associations with the participants’ age. A systematic review of different studies disclosed that higher age was associated with an increasing rejection of resuscitation measures [67]. Compared to that, research on people’s attitudes on technology usage in end-of-life situations hardly exists. It is so far not known to what extent concrete prospective decisions related to technology usage in end-of-life situations are impacted by demographic and individual characteristics, such as people’s age and health status. Therefore, the third research question was the following:

- RQ3: do decisions regarding using assistive technology in end-of-life situations differ depending on the participants’ age and health status?

### Research gap, objective, and aim of the study

Considering the introduced literature and the derived research questions, it is not clarified so far whether, and to what extent, age and health status as individual factors impact perceptions of aging, acceptance of using assistive technology in older age, as well as the assessment of end-of-life situations.

Addressing these research gaps, the associated aim of the present study was to examine the potential influence of the participants’ connected age and health status (as a four-field matrix) on their perceptions of aging, their perceptions of using assistive technology in old age, as well as end-of-life-decision-making. Thereby, the perspectives of younger and older participants were of equal importance and should be compared, as the age-related perceptions of younger people have not only the potential to shape but might even considerably influence the adoption of assistive technologies and respective decisions in their later stages of life. To reach this aim, an online survey was conceptualized focusing on individuals covering the whole adulthood span and characterized by diverse health conditions as an important expression of biological age. The underlying research questions were derived based on the research state presented above. Beyond that, the last step of the analysis connects the three research questions by investigating the relationships between all investigated constructs.

## Methods

In this section, the methodological approach including the applied online survey is introduced, followed by short descriptions of data analysis procedures and the sample. Finally, the applied group segmentation is presented.

### Approach and online survey

Based on the previous literature analysis and preceding studies described in detail in [21, 32], an online survey was developed aiming for an investigation of the perceptions of aging, acceptance of using assistive technologies in old age, and end-of-life-decisions, targeting equally participants of all ages and with diverse health conditions. In the beginning, the participants were welcomed and shortly introduced into the topic of an aging society and the development of assistive technology to support life in old age.

**Fig. 1:**
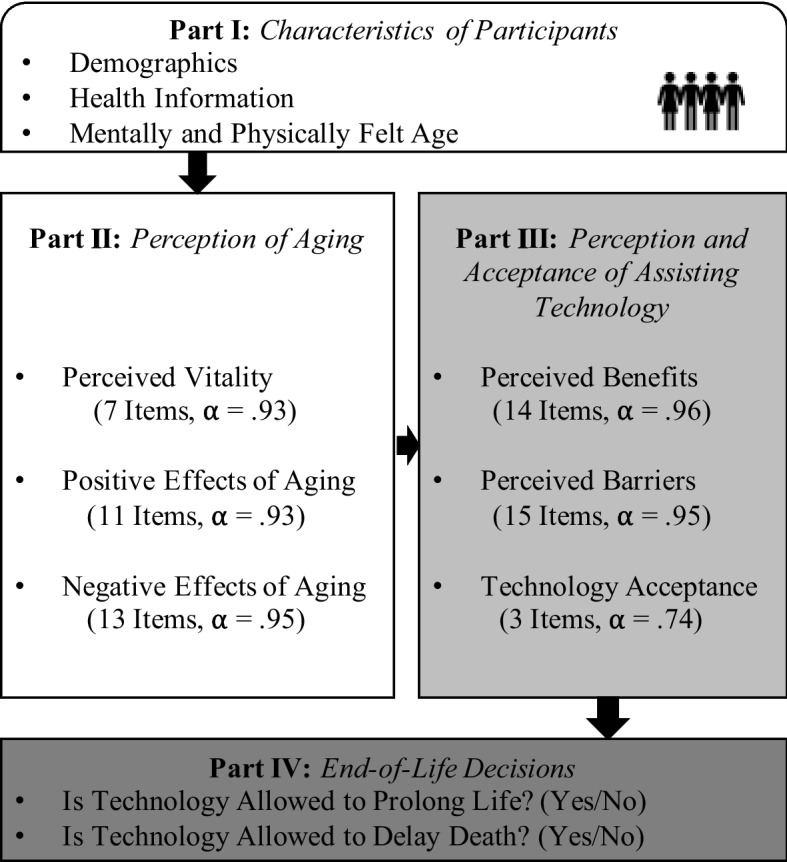
Overview of the online survey design (for more details see Appendix 1)

The survey consisted of four parts presented in Fig. 1. In the first part, the participants were asked to share their individual characteristics, starting with indicating demographic information, i.e., their age, gender, highest educational level, and living situation. Subsequently, the participants indicated if they suffer from a chronic health condition or an impairment (answer options: yes/no). Here, they were able to comment on their conditions and impairments on an optional basis. Frequent examples at this point were chronic heart failure, diabetes, or osteoarthritis. To find out their perceived a) mental and b) physical age, participants indicated whether they felt younger or older than their actual age. A scale from 0 to 100 was used to provide a finely granulated assessment, with 50 representing the current actual age and the scale ends "0 = much younger" and "100 = much older" than the actual age.

Following these details, the participants were introduced to the topic of aging within the second part of the survey. In the next step, they evaluated age-related aspects and assessed their perceived vitality using seven items (Cronbach’s ⍺ = 0.93; item examples: “I feel vivid and vital” or “I look forward to every new day”; adapted from [35]). Further, the participants evaluated their attitudes toward aging by an assessment of the potential positive and negative effects of aging. For this purpose, the participants evaluated 11 items referring to possible positive effects of aging (⍺ = 0.93; item examples: “Aging means to me that I can still have a lot of fun in life” or “Aging means to me can still be very useful for society and my family due to my high life experience” based on [12], extended and adapted from [21, 32, 55]). Afterwards, the respondents evaluated 13 aspects regarding potential negative effects of aging (⍺ = 0.95; item examples: “Aging means to me that I am a burden to my family” or “Aging means to me to be less independent” based on [12], extended and adapted from [21, 32, 55]). The evaluation of these three constructs took place using on six-point Likert scales (reaching from”1 = I totally disagree” to”6 = I totally agree”) and all assessed items and their origin are presented in Appendix 1.

Next, the participants were introduced to the development of assistive technologies and to their potential of providing support and assistance in old age. They should empathize with a situation, in which they need care and the use of assistive technology could facilitate their everyday life, e.g., by immediately reporting emergencies and accidents such as falls or by reminding them of daily routines.

Within the third part of the survey, the participants were first asked to assess different potential benefits of assistive technology by using 14 items (α = 0.96; item examples: “Increase in independence (autonomy)” or”Fast reactions in emergencies” based on and adapted from [21]). Then, they evaluated 15 different potential barriers to using assistive technology (α = 0.95; item examples: “Insights into personal health data for unauthorized persons” or “Replacing human care by technology”, based on and adapted from [21]). In addition, the participants were asked to assess three different items measuring the acceptance of using assistive technology in older age (α = 0.95; item example: “I can imagine using assistive technology”, based on [46, 47]). For all these evaluations, also six-point Likert scales were used. All assessed items as well as their origins are presented in Appendix 1.

The last part of the survey connected the topics of the second and the third part of the survey: The survey familiarized the participants with severe health situations in old age in which using assistive technology can be particularly useful in such contexts. In this context, however, it was also clarified that it is questionable what technology is allowed to do in such severe health situations. On this basis, respondents judged in two separate questions whether technology is allowed to a) prolong life and b) delay death. For these questions, the nominal answer options “yes/no” were used and the participants were asked to make explicit decisions.

Finally, the participants had the opportunity to give feedback on the topic and the survey itself.

### Data acquisition, data cleanding and analysis

To reach a representative sample regarding age and to enable group comparisons of healthy and persons with chronic conditions, the service of an independent market research institute was used for data collection. For data acquisition, quotas on age were defined with three almost equally distributed groups (i.e., < 40 years, 40–60 years, and 60 + years) as well as quotas on health status comparing healthy individuals with those with a chronic condition. Hence, it was aimed at reaching a sample being balanced and covering all age groups and diverse health conditions. All participants were paid for their participation.

Overall, *N* = 1385 participants consented to participate in the study. As only complete data sets can be used for statistical analyses, in the beginning, incomplete and canceled data sets were excluded from the database. In this regard, *n* = 718 participants cancelled the survey within the first 60 s of processing time. In addition, participants with very short processing times (less than the median of the processing time − 30%) were excluded (*n* = 94). Further, participants younger than 18 years were excluded from the data set as the study aimed at an investigation of the whole adulthood based on a broad and diverse sample (*n* = 3). Overall, *n* = 815 data sets were excluded from further analysis, primarily due to too short processing times or cancellation of the survey. Analyses showed that – not surprisingly – those data sets would also have been excluded due to incompleteness and inadequate data quality in terms of inconsistencies in response behaviour. Discussions with the market research institute revealed that proportion between 50 and 70% of excluded data sets represent a common ratio depending on the defined quotas.

As preparation for the data analysis, negative items were re-coded, all scales were tested for reliability, and overall scores were calculated. For the statistical analysis of the influence of the participants’ age and health status, the participants were divided into four groups (see section “Age and health status segmentation”). Using analyses of variance (ANOVA) and chi-quadrat tests (χ^2^), influences of the interaction of age and health status on the participants’ perception of aging, acceptance/perception of assistive technologies, and life-end decisions were investigated. Thereby, partial eta squared (η^2^) was calculated for effect sizes. To identify specific differences between the four groups, Tukey’s post-hoc tests were applied. Significant group differences are presented using square brackets within the diagrams in addition to the respective significance level. In the following, means (M) and standard deviations (SD) are reported for descriptive statistics and the level of statistical significance (p) was set at the conventional level of 5% (*p < 0.05; **p < 0.01).

### Sample

Overall, *N* = 570 participants filled out the survey completely. Data were collected in Germany, and the participants took an average of 20 min to complete the survey. All participants indicated to come from and currently live in Germany, whereas the distribution over Germany (North, East, South, West) was almost on an equal level.

The mean age of the participants was 47.7 years (SD = 16.3; min 19; max 84; median 50) and gender was balanced within the sample: 273/570 (47.9%) females, 297/570 (52.1%) males. The educational level was, on average, rather high because 21.8% of the sample (124/570) reported a high level holding a university degree and 55.1% (314/570) a medium level holding a university entrance qualification or a completed apprenticeship. 23.2% (132/570) reported a low educational level (secondary school degree). Regarding their living situation, a third of the participants indicated to live alone (34.7%, 198/570), while 64.6% (368/570) live together with another person, and *n* = 4 participants did not indicate their living situation (0.7%). Asked for health-related information, more than half of the participants reported to suffer from a chronic health condition (61.8%, 352/570). In this regard, the participants mentioned a broad range of chronic health conditions reaching from mild (e.g., allergies, asthma), over typical age-related (e.g., arthrosis, diabetes, hypertension) to severe chronic health conditions (such as morbus Parkinson, multiple sclerosis, COPD). Overall, most of the mentioned clinical patterns referred to typical chronic health conditions (e.g., diabetes, hypertension), whereas impairments were only sporadically mentioned (e.g., tetraparesis). If "chronically ill" is mentioned in the further course, this therefore refers primarily to participants with a chronic health condition. Only a small proportion of the participants indicated to need assistance and care in their everyday life (12.3%, 70/570). To enable comparisons between young and old as well as healthy and chronically ill participants, a four-field scheme was used. In this way, the participants were divided in four groups differing by their age and health status. The segmentation process is explained in the next section.

### Age and health status segmentation

Aiming for an intentional investigation and categorial examination, we categorized four groups depending on the participant ‘s age and health status. Based on the participants’ self-assessed indications if they suffer from a chronic illness, they were classified as healthy or chronically ill persons. Participants were definded to be “healthy” persons, if they indicated to have “no chronic illnesses” and that they do “not depend on assistance and care” in their everyday life. Vice versa, if the participants indicate to suffer from a chronic illness, they were classified as “chronically ill”. Depending on the reported type of their chronic illness, the co-authoring medical experts checked the indicated chronical illnesses and decided retrospectively if the persons were correctly identified to be “chronically ill” persons. Regarding age, a cut-off of 50 years was chosen (median-split) to separate young (< 50 years) from old participants (≥ 50 years). Besides statistical reasons (median-split), this cut-off was chosen as already during the 6th decade people approach towards the brink of calendar aging being old, representing a turning point when people start thinking more about their lives and possible preventive measures for life in older age [68]. Accordingly, four groups were segmented: “younger healthy”, “younger chronically ill”, “older healthy”, and “older chronically ill”. A comparison of the four groups revealed significant differences – not surprisingly – regarding age (F(3,569) = 458.4; *p* = 0.000). Thereby, the results showed that the “younger chronically ill” participants were a bit older (M = 35.7; SD = 10.0) than the “younger healthy” participants (M = 31.5; SD = 9.5), while the “older healthy” (M = 61.8; SD = 8.6) and the “older chronically ill” (M = 60.8; SD = 7.3) groups did not significantly differ regarding their age. The other relevant demographic characteristics of the groups are shown in Table 1. Furthermore, the four groups also differed regarding their distributions of gender. As depicted in Table 1, both young groups contained slightly higher proportions of female than male participants, while both older groups contained more males than female participants. The four groups did not differ regarding their educational level and living situation.

**Table 1:** Characteristics of the segmented groups

Variable	Younger healthy (*n* = 123)	Younger chronically ill (*n* = 158)	Older healthy (*n* = 95)	Older chronically ill (*n* = 194)	Statistics of Difference
*Gender (w/m)*	60.2% (74/123)	52.5% (83/158)	35.8% (34/95)	42.3% (82/194)	χ^2^(3,570) = 16.8; *p* = .001
	39.8% (49/123)	47.5% (75/158)	64.2% (61/95)	57.7% (112/194)	
*Education (low/medium/high)*	24.4% (30/123)	22.2% (35/158)	22.1% (21/95)	23.7% (46/194)	n.s. (*p* = .083)
	54.5% (67/123)	62.0% (98/158)	42.1% (40/95)	56.2% (109/194)	
	21.1% (26/123)	15.8% (25/158)	35.8% (34/95)	20.1% (39/194)	
*Living Situation (alone/not alone)*	34.1% (42/123)	32.9% (52/158)	36.8% (35/95)	35.6% (69/194)	n.s. (*p* = .916)
	65.0% (80/123)	66.5% (105/158)	62.1% (59/95)	63.9% (124/194)	
	(*n* = 1; missing)	(*n* = 1; missing)	(*n* = 1; missing)	(*n* = 1; missing)	

**Fig. 2:**
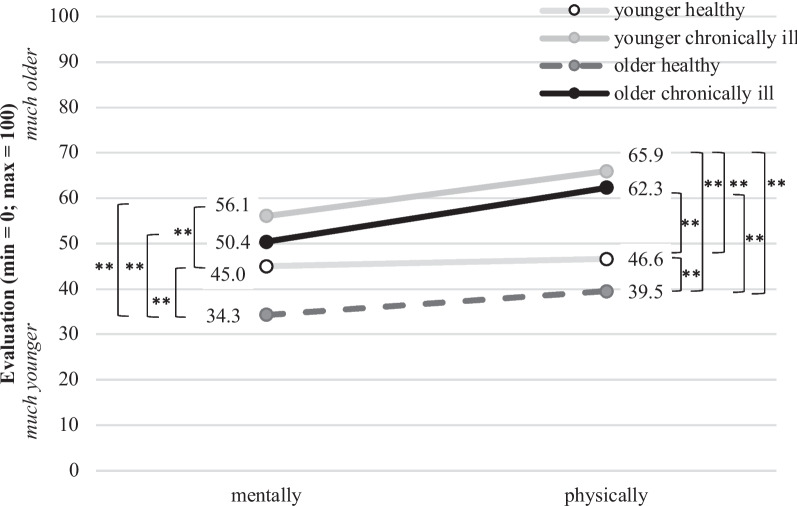
Evaluation of the perceived health status (mentally and physically) for the segmented groups (asterisks (**) indicate a significance level of *p* < .01)

As a first group-describing result, the participants were asked to judge their perceived health status and to indicate if they feel a) mentally and b) physically younger or older in relation to their chronological age. The results of the participants’ evaluation are presented in Fig. 2.

The results revealed significant group differences for both, the mental (F(3,569) = 23.7; *p* = 0.000) and physical (F(3,554) = 58.7; *p* = 0.000) perceptions of the participants’ health status. In comparison to the other groups, the “older healthy” group indicated to feel mentally (M = 34.3, SD = 18.4) as well as physically (M = 39.5, SD = 17.8) significantly younger than their chronological age. According to the scale midpoint (M = 50), the “younger healthy” group reported feeling mentally (M = 45.0, SD = 19.8) and physically (M = 46.6, SD = 19.3) slightly younger than their real age. This group differed significantly from the groups “older healthy” as well as the “younger chronically ill”.

In comparison, the groups of “younger chronically ill” (M = 56.1, SD = 20.2) and “older chronically ill” (M = 50.4, SD = 22.4) respondents exposed similar evaluation patterns indicating to feel mentally as old as their actual age, while tending to feel physically older than their chronological age (“older chronically ill”: M = 62.3, SD = 19.1 and “younger chronically ill”: M = 65.9, SD = 16.7). Hence, the results showed the tendency that almost independently from their age, persons with chronic conditions felt older than healthy persons.

## Results

In the following, the results of the study are described. To give an impression of the attitude towards aging that is assumed to have an impact on the adoption of assistive technologies in everyday life, we present at the outset of this section the results of our study respondents’ perceptions of aging-related constructs. In a next step, it is investigated whether, and to what extent, the four groups differ in their perceptions and acceptance of using assistive technology in old age. In a final step, the evaluations of life-end decisions are presented.

### Attitudes towards Aging (RQ1)

Within the evaluation of aging-related constructs, the participants assessed their vitality in their current life situation. But they also evaluated possible positive as well as negative effects of aging. For these three constructs, the group-related results are described regarding the overall scores.

#### Vitality

**Fig. 3:**
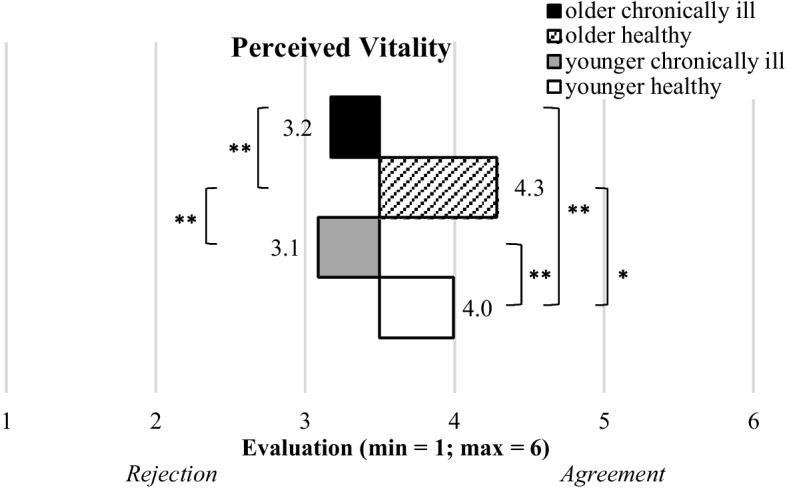
Descriptive statistics of perceived vitality of the segmented groups (bars represent means and asterisks indicate the significance level: * *p* < .05; ** *p* < .01)

The results, as depicted in Fig. 3, revealed that the four groups significantly differed concerning their evaluation of their perceived vitality (F(3,569) = 43.8; p = 0.000; η^2^ = 0.188). The “older healthy” (M = 4.3, SD = 0.9) as well as the “younger healthy” (M = 4.0, SD = 0.9) respondents showed the highest evaluations indicating an energetic, vivid, and vital feeling within their current life situations. In contrast, the participants with chronic conditions (“older”: M = 3.2, SD = 1.1 and “younger”: M = 3.1, SD = 1.1) slightly rejected items on the vitality scale (M < 3.5), clearly indicating their perceptions of lower vitality. In more detail, these groups rejected to feel energetic, vivid, and vital and showed a higher agreement to feel powerless.

#### Positive and negative effects of aging

**Fig. 4:**
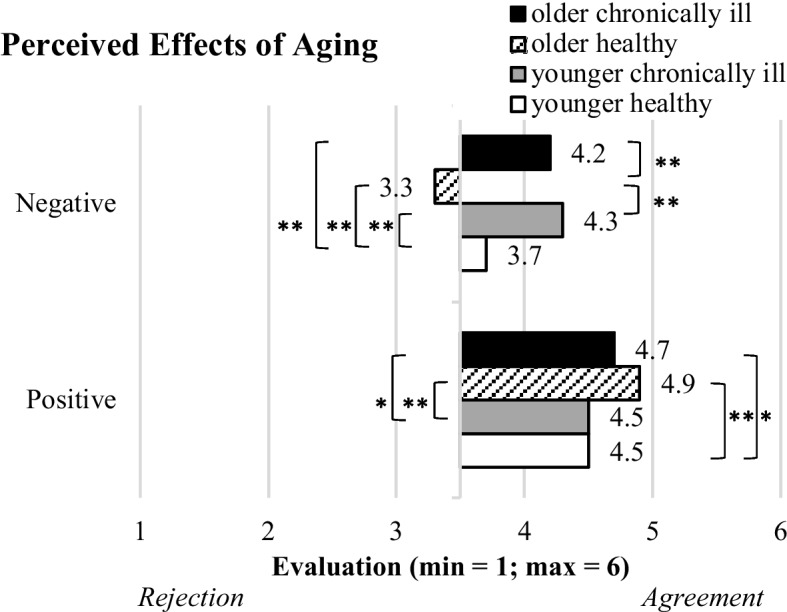
Descriptive statistics of positive and negative effects of aging for the segmented groups (bars represent means and asterisks indicate the significance level: *p < .05; **p < .01)

The participants’ evaluation of potential positive and negative effects of aging is illustrated in Fig. 4, where significant differences for both, the positive and negative effects of aging, were found. As to positive effects, analysis of variance revealed significant but comparably weak differences (F(3,569) = 6.9; *p* = 0.000; η^2^ = 0.035). The “older healthy” (M = 4.9, SD = 0.7) and the “older chronically ill” (M = 4.7, SD = 0.8) groups showed slightly higher evaluations of the potential positive effects of aging compared to the “younger healthy” (M = 4.5, SD = 0.8) and the “younger chronically ill” (M = 4.5, SD = 0.8) groups. Considering the evaluations of negative aging effects, significant and even more distinct differences were found too (F(3,569) = 24.4; *p* = 0.000; η^2^ = 0.114). Here, the participants in the “younger chronically ill” (M = 4.3, SD = 1.0) and the “older chronically ill” (M = 4.2, SD = 0.9) groups confirmed the negative effects of aging, while lower agreements resulted for the “younger healthy” (M = 3.7, SD = 1.0) participants and for the “older healthy” (M = 3.3, SD = 1.0) group. Overall, both older groups showed higher means of the positive effects of aging compared to both younger groups, while participants in the groups with chronic health conditions indicated higher confirmations of the negative effects of aging compared to the individuals of both healthy groups.

### Attitudes towards assistive technology (RQ 2)

As assistive technologies have the potential to support life in older age, the participants’ acceptance as well as perceptions of using assistive technology in their everyday life were evaluated. In the following, the results of perceived benefits and barriers as well as the indicated acceptance of assistive technologies are analyzed.

#### Perceived benefits and barriers

**Fig. 5:**
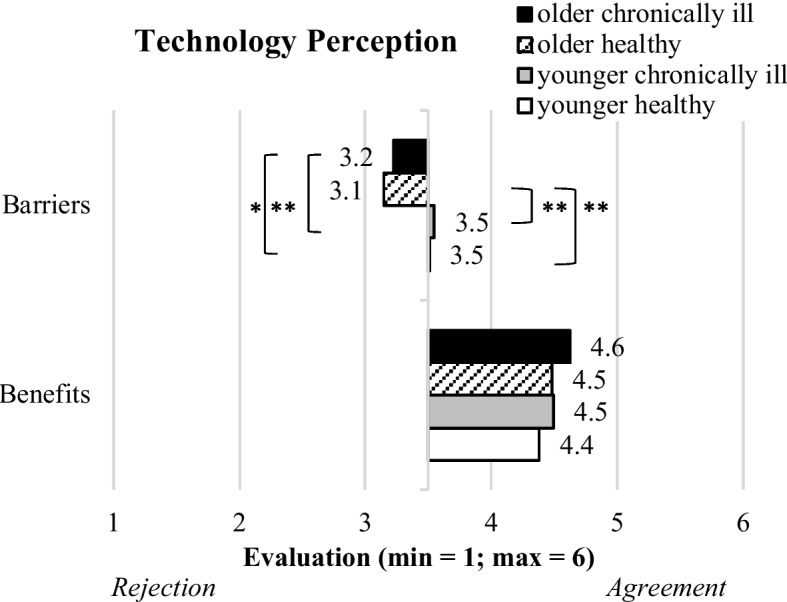
Descriptive statistics of perceived benefits and barriers of assistive technology for the segmented groups (bars represent means and asterisks indicate the significance level: *p < .05; **p < .01)

The results regarding the perception of benefits and barriers are presented in Fig. 5. The evaluation of perceived benefits did not differ significantly in the four study groups (F(3,559) = 1.5; p = 0.225). All four groups congruently agreed on the perceived benefits of using assistive technology: “older chronically ill” (M = 4.6, SD = 0.9), “older healthy” (M = 4.5, SD = 0.9), “younger chronically ill” (M = 4.5, SD = 0.8), and “younger healthy” (M = 4.4, SD = 0.8).

As to the perception of barriers, significant but weak differences resulted for the study groups (F(3,569) = 5.9; *p* = 0.001; η^2^ = 0.018). Thereby, both young groups (“younger healthy”: M = 3.6, SD = 1.0 and “younger chronically ill”: M = 3.6, SD = 1.1) showed neutral evaluations, while the respondents of the “older chronically ill” (M = 3.2, SD = 1.0) as well as the “older healthy” (M = 3.1, SD = 1.0) group tended to slightly reject the barriers of using assistive technology in older age.

Overall, all groups confirmed the benefits of using assistive technology in older age, while the barriers were slightly more pronounced in the younger compared to the old groups.

#### Acceptance of assistive technology

**Fig. 6:**
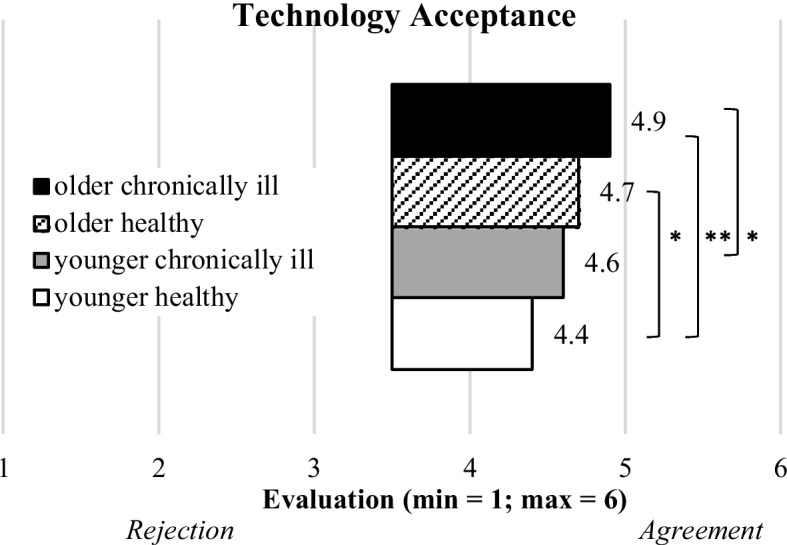
Descriptive statistics of acceptance of assistive technology for the segmented groups (bars represent means and asterisks indicate the significance level: *p < .05; **p < .01)

For the acceptance of using assistive technology in old age, also significant but weak differences were identified (F(3,569) = 4.8; *p* = 0.003; η^2^ = 0.025) and the results are shown in Fig. 6. All groups accepted assistive technology and the highest evaluations were found for the “older chronically ill” group (M = 4.9, SD = 0.9). Nevertheless, the other study groups also showed comparably high agreements on the adoption of using assistive technology: “old and healthy” (M = 4.7, SD = 0.9), “younger chronically ill” (M = 4.6, SD = 1.0), and “younger healthy” (M = 4.4; SD = 1.0).

### End-of-life decision-making (RQ 3)

In the final step of the survey, the participants evaluated what technology is allowed to do in end-of-life situations. In more detail, they assessed whether technology is allowed to prolong life and, vice versa, whether it is allowed to delay death. As the answer options were “yes” or “no” for these questions, chi-quadrat tests were calculated.

**Fig. 7:**
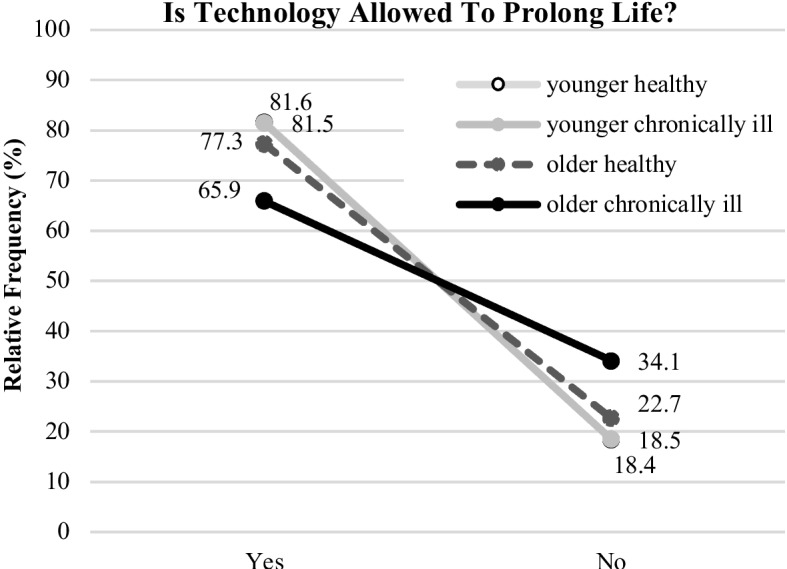
Opinions on life-end decisions (“…prolong life”) for the segmented groups (illustrated are relative frequencies in %)

For the first question (“Is technology allowed to prolong life?”), the results are shown in Fig. 7 and revealed significant differences for the four groups (χ^2^(3,532) = 14.2; p = 0.003). Highest and identical agreements were found for the younger groups (“healthy” *yes*: 81.6% (93/114), *no*: 18.4% (21/114); “chronically ill”: *yes*: 81.5% (123/151), *no*: 18.5% (28/151)). The “older healthy” group showed similar results (*yes*: 77.3% (68/88); *no*: 22.7%(20/88)). In contrast, individuals categorized as the “older chronically ill” group agreed significantly less (*yes*: 65.9% (118/179)) and rejected more (*no*: 34.1% (61/179)) the permission for the technology to prolong human life.

**Fig. 8:**
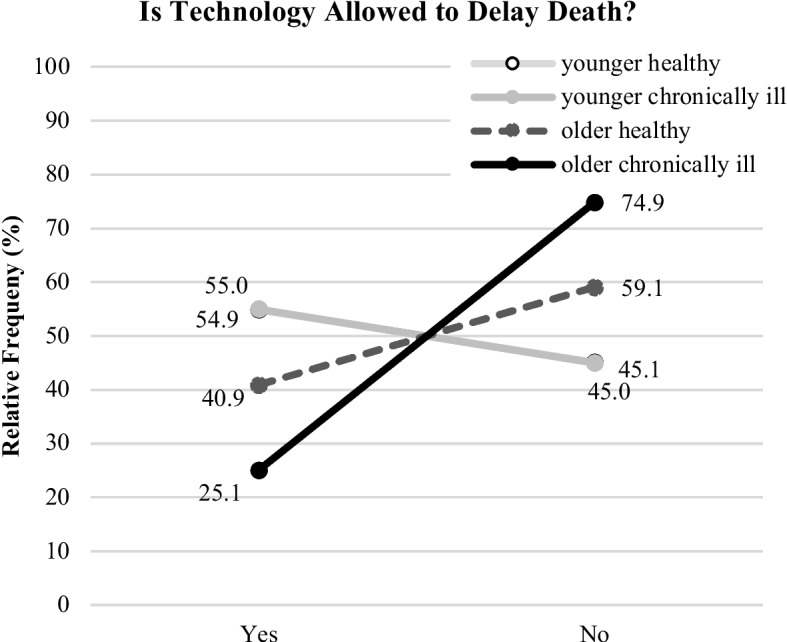
Opinions on life-end decisions (“…delay death”) for the segmented groups (illustrated are relative frequencies in %)

Related to the question “Is technology allowed to delay death?”, the statistical analysis revealed significant and even more distinct results (χ^2^(3,531) = 38.8; p = 0.000). The evaluations of “younger healthy” (*yes*: 54.9% (62/113); *no*: 45.1% (51/113)) and “younger chronically ill” participants (*yes*: 55.0% (83/151); *no*: 45.0% (68/151)) were again comparable and tended more to agree on this question. In contrast, the “older healthy” group decisively stronger rejected to allow technology delaying death: *yes*: 40.9% (36/88); *no*: 59.1% (52/88). However, the most distinct evaluation pattern was identified for the “older chronically ill” group of participants: with a rejection of 74.9% (134/179) (*yes*: 25.1% (45/179)), this group voted against the option of the technology to delay death. The outcomes are depicted in Fig. 8.

### Relationships between investigated constructs (RQ1-RQ3)

In the first step, correlations analyses revealed that the aging-related perceptions (RQ1) are only partly interrelated. Perceived vitality correlated positively with the positive effects of aging (*r* = 0.31, *p* < 0.01) and negatively with the negative effects of aging (*r* = -0.38; *p* < 0.01), while the perceptions of positive and negative effects of aging were not connected (*r* = 0.07; n.s.). In the second step (RQ2), technology acceptance correlated strongly with perceived benefits (*r* = 0.62; *p* < 0.01) and to a lesser extent with perceived barriers (*r* = -43; *p* < -01), whereas perceived benefits and perceived barriers showed a weak interrelation (*r* = -0.21; *p* < 0.01). Focusing on the end-of-life situations (RQ3), both statements, “… to prolong life” and “… to delay death”, revealed to be weakly related (*r* = 0.32; *p* < 0.01).

Beyond that, the results revealed relationships between the constructs. Considering aging- and technology-related perceptons (RQ1 & RQ2), the positive effects of aging strongly correlated with perceived benefits (*r* = 0.59; *p* < 0.01), perceived barriers (*r* = -0.14; *p* < 0.01), and technology acceptance (*r* = 0.47; *p* < 0.01). To a lesser extent, the negative effects of aging related to perceived benefits (r = 0.23; *p* < 0.01), perceived barriers (*r* = 0.36; *p* < 0.01), and technology acceptance (*r* = 0.11; *p* < 0.01). In contrast, significant relationships between perceived vitality and the technology-related evaluations have not been disclosed (n.s.). Investigating relationships between aging-related perceptions and the evaluation of life-end decisions (RQ1 & RQ3), perceived vitality represented the own construct being weakly negatively connected with the statement “…prolong life” (*r* = -0.13; *p* < 0.01). Regarding the technology-related perceptions, all constructs revealed weak correlations with the statement “…prolong life”: perceived benefits (*r* = -0.17; *p* < 0.01), perceived barriers (r = 0.14; *p* < 0.01), and technology acceptance (*r* = -0.16; *p* < 0.01). Interestingly, the second life-end decisions statement “…delay death” was neither related with aging- nor technology-related perceptions (n.s.). Summarizing the results of the correlation analyses, these relationships as well as the effects of age and health status as individual factors (see Sects. 3.1–3.3) highlight the necessity of holistically investigating technology acceptance in older age, taking individual factors, aging-related perceptions, technology-related perceptions, and life-end decisions into account.

## Discussion

This section presents a discussion of this study’s key findings and a first derivation of practical, medical implications of how the results can be used and applied in clinical practice and research.

### Key insights and their meanings

Regarding age-related perceptions (RQ1), findings of our study show that diseased persons indicated to feel older than healthy persons, independently from their chronological age. These results confirm previous findings [69, 70], in which objective physical health conditions as well as subjective health perceptions, i.e., self-rated health status, explained the largest proportions of variance, indicating that poorer health contributes to feeling older. In addition to previous findings, this study revealed that persons with chronic conditions – independently from their chronological age – showed lower vitality evaluations and indicated to feel less vivid and vital compared to healthy participants. We can thus assume that individuals with chronic health conditions are more familiar with the burden and are accordingly more experienced regarding restrictions within day-to-day life, leading to lower vitality evaluations.

The participants’ age and health status also significantly influenced their perceptions of aging. Here, the positive aspects of aging were rated higher by older than the younger participants, while the negative aging effects were more pronounced by chronically ill participants as opposed to the healthy respondents (independent from their age). In parts, these results confirm previous research [59] that revealed higher evaluations of positive aspects of aging by older participants compared to younger generations. It is assumed that participants who are closer to the retirement age relativize wishes and needs and, thus, perceive and appreciate more benefits and positive aspects of aging compared to younger people. In contrast, the results regarding the evaluations of negative aspects of aging go beyond previous findings. The negative framing of aging in persons with a poorer health status might be explained by higher fears and concerns regarding living with a (severe) age-related disease in the future. Here, we hypothesize that the type and the severity of the disease might influence the evaluations of aging and well-being (see suggestions for future work in Sect. 4.3). Besides the chronological age and health status (based on clinical pictures), subjectively perceived age as well as health status also represent relevant factors potentially influencing the perceptions of aging. Based on previous research in this field [69, 70], we assume that people who feel older than their chronological age feel less vital and have a more negative attitude towards aging than individuals who estimate themselves younger than they actually are. Future analyses could investigate whether people who feel younger than their real age perceive aging more positively compared to people who feel older than their real age. A similar subjective assessment could also be used for the perceived health status comparing people with a perceived good and poor state of health regarding their perceptions of aging (e.g., vitality, effects of aging).

While there were clear-cut differences concerning the age-related perceptions across groups (RQ1), the benefit perceptions (RQ2) of using assistive technology in older age did not vary depending on age and health status. However, it should be noted that the assessments were comparably high, showing that people acknowledged the surplus the medical assistive technology can bring for them. When it comes to the perceived barriers to using assistive technologies in older age, lower agreements were found by older compared to younger participants. Hence, the barriers to using assistive technologies were less relevant for older than younger people. These relationships (i.e., no effects regarding perceived benefits, the effect of age on perceived barriers) confirm the findings of previous research [51, 71], in which these patterns were identified for different technology applications and care contexts. Finally, this study revealed that the older and participants with chronic conditions admit the highest acceptance of using assistive technologies in older age. Whereas previous research identified the participants’ health status to be relevant for technology acceptance to some degree [55, 56], this study showed the highest technology acceptance among older and chronically ill participants. We assume that the felt necessity of using assistive technologies is the highest for this specific user group and, accordingly, this group shows the highest acceptance evaluations. Besides the chronological age and clinical pictures (as mentioned above), the subjectively perceived age as well as the perceived health status represent factors that are potentially influencing the perceptions and acceptance of assistive technologies. Based on the comments of our participants, we assume that people feeling older than their real age or with a poor perceived state of health, who are maybe even healthy from the medical point of view, tend to be more open to using and adopting assistive technologies due to their perceived need for support and assistance. Future studies should therefore focus on these relationships and compare people who feel younger/older than their actual age with a good/poor perceived state of health referring to their evaluations of benefits, barriers, and acceptance of using assistive technologies in older age.

The most striking differences and novel insights were found for the end-of-life decisions (RQ3). Beyond identified influences of age on the end-of-life decisions (e.g., [66, 67]), this study identified different decision patterns depending on the participant’s age and health status. In addition, the results showed different evaluation patterns for technology being allowed to prolong life in comparison to technology being allowed to delay death, even though in medical terms this means the same. That technology is allowed to prolong life was strongly confirmed by the younger “healthy” and “chronically ill” participants likewise (80% “yes”), and the “older healthy” participants have also opted for it. Only the „older chronically ill” group showed significantly lower agreement in this context (65% “yes”). Compared to the other study groups, the older individuals and participants with chronic conditions are more reserved to accept using assistive technology in severe health situations. This finding is of utter importance: From a social and ethical point of view, it shows that medical assistive technology is not the “all-encompassing solution” for the care of older adults, even if this might be a good option from a technical and economic perspective. It rather seems to be the case that the perceived usefulness of this technology is also a function of age and health. Moving to the question, if technology is allowed to delay death from the perspective of participants, we found even more distinct evaluation patterns. Again, it was the younger persons who had a comparable view: Independently of their health status, 56% agreed to technology delaying death; the “older healthy” participants were more reluctant in this regard (40% “yes”). The older participants with chronic conditions reacted – in contrast – in the opposite way. Only 25% of this group would allow technology to delay death. Here, limits of acceptance show up: Whenever technology helps to live independently and in dignity, it is welcome, but when used to prolong life, then older and chronically ill persons decline its usage. Instead, this group wished to decide themselves in end-of-life decisions, even more than all other investigated user groups.

### Practical and medical implications

Our findings place the matter of acceptance of assistive technologies in the context of autonomy and its endangerment due to either advancing chronological age or biological age, the latter more closely reflecting the individual destiny than the former (see Sect. 1.1). Permitting the assistance of technologies is rather triggered by unpredictable functional impairments, such as chronic diseases, than by advancing chronological age with its inevitable shrinking of further life expectancy. The direct self-concern increases the acceptance of assistive technologies as long as it does not affect the existential question of life and death. Assistive technologies used to overcome barriers to functionality, activities, and participation are appreciated as measures for enhancing self-defined autonomy. Conversely, assistive technologies in the context of end-of-life situations appear afflicted with a too-invasive intrusion into personality and might therefore run counter to the notion of autonomy.

In the everyday life of geriatrics and nursing, technical assistance will become more and more important for the patient, the caregivers, and all persons authorized to manage the patient’s affairs. The decision to employ assistive technology in geriatrics and nursing cannot be broken down into a dichotomous “yes or no “-question. It has to be backed up by a specific refinement as to how a certain assistive technology can a) compensate for specific impairments of functionality, b) enhance activities and participation, as well as c) finally strengthen autonomy. The range and need of assistive technologies differ – depending on the biological age – both interindividually and longitudinal-intraindividually. In addition, there is also an overlapping array between enhancement of autonomy and prolongation of a lifetime in which the respective objective of assistive technologies cannot be clearly assigned yet. Notwithstanding already existing geriatric concepts (such as frailty and multimorbidity), a reliable and sustainable comprehensive mapping poses therefore a laborious challenge. Here, an early and periodic assessment is even more important to properly adapt and individualize technological measures step by step.

### Conclusion, limitations, and future research

This study revealed novel insights concerning the impact of the participant’s age and health status on attitudes towards aging and using assistive technology in older age and care. Thereby, the health status was more decisive for aging-related perceptions compared to the chronological age, while technology-related perceptions (i.e., perceived barriers and acceptance) were slightly impacted by either the chronological age or the participants’ health status. Compared to that, the most striking differences were identified about the end-of-life decisions, revealing older adults with chronic conditions to have a more restrained attitude towards technology prolonging life or delaying death compared to the older healthy persons and younger participants.

Besides these insights, the applied approach also entails some methodological and content-related limitations, that should be considered in future research. Starting with this study’s participants, a large sample of participants from all ages and with different health conditions took part in the study. For data acquisition, quotas were defined with regard to age and health status leading to equal distributions regarding all age groups and health conditions. Nevertheless, it would be valuable trying to reach even more participants being older than 65/75/85 years of age to analyze age-related differences in more detail. For this purpose, in addition to the online survey approach paper and pencil questionnaires should be used to reach these participants to a broader extent. The latter becomes even more important since data acquisition was conducted exclusively online and therefore the representativeness of the sample is restricted to the subset of the population being able to complete online surveys.

A further methodological limitation lies in dichotomizing age and health status as analyzed individual parameters. Of course, this approach represents a kind of an artificial classification; however, it enabled to categorically investigate the effects of combined age and health status groups of participants detecting relevant impacts on age-related as well as technology-related perceptions and life-end decisions. Future approaches should also integrate these variables as contionous variables and should – beyond that – consider the type and severity of the chronical illnesses as these parameters could influence the attitudes towards aging and perceptions of using technology in older age and care as well. Therefore, future studies should consider a medical categorization of (age-related) diseases and analyze their potential impact on the attitudes towards aging, technology-related perceptions, as well as end-of-life decisions.

A further sample-related limitation refers to the cultural background of the participants. In the present study, all participants came from one single country – Germany. As perceptions, whishes, beliefs, and opportunities vary across different cultures, backgrounds, and origins, future studies should focus on cross-cultural comparisons by investigating the participants’ race and ethnicity in more detail.

Another limitation refers to the scenario-based approach applied in this study. According to the well-known gap between attitudes and behavior [72], we should bear in mind that the end-of-life decisions in this study presumably differ from decisions in severe real-life contexts, in which not only the perspective of the person concerned but also perspectives of family members, care personnel, and specialist physicians are implicated. As to the scenario-based approach, the fact that the participants did not receive specific information before the assessments, i.e., no explicite descriptions of the specific situation or aging context, should also be mentioned as a limitation too. Here, the aim was to obtain a generic picture and the unbiased opinions of the participants, as previous research revealed that the way of information presentation shapes the subsequent evaluations, e.g., using detailed scenarios differing in their necessity of care [71]. Future studies could investigate how positively or negatively described aging scenarios influence the evaluations of aging-related and technology-related perceptions.

### Supplementary Information

**Additional file 1.** Overview of all assessed constructs and their respective items.

**Additional file 2.**

## Data Availability

All data generated or analyzed during this study are included in this published article and its supplementary information files.
