# Supplementary material for: How age and health status impact attitudes towards aging and technologies in care: a quantitative analysis
Source: BMC Geriatr. 2024 Jan 3;24:9. doi: 10.1186/s12877-023-04616-4 (PMC10765835; doi:10.1186/s12877-023-04616-4)
Supplement: Supplementary file 1 — Additional file 1. Overview of all assessed constructs and their respective items. [file 12877_2023_4616_MOESM1_ESM.pdf]

Appendix 1: Overview of all assessed constructs and their respective items.

Construct	*Measurement*	**Origin**	*Cron-bach's α*
**Perceived Vitality**	1. I feel alive and vital.<br>2. I feel powerless.<br>3. Sometimes I feel so alive that I could uproot trees.<br>4. I have a lot of energy and zest for action.<br>5. I look forward to every new day.<br>6. Almost always I feel lively and awake.<br>7. I feel full of energy.	Adapted from Ryan & Frederick (1997) to measure perceived vitality in more detail	.93
**Positive Effects of Aging**<br><br>*"I think in older age, I …*	1. ... am far more mobile and independent than 20 years ago".<br>2. ... thanks to modern technology can live predominantly independently."<br>3. ... have to keep up to date with the latest developments in order to keep up to date."<br>4. ... can still have lots of fun in life."<br>5. ... can maintain good health with plenty of exercise and careful nutrition."<br>6. ... can overcome adversities better through personal experience."<br>7. ... must be well informed and open to technical innovations."<br>8. ... must be flexible and adapt to developments and changes."<br>9. ... must continue to take care of social contacts."<br>10. ... can still be very useful for society and the family due to my life experience".<br>11. ... has much more time for things you always wanted to do."	Based on analyses of Wurm et al., 2007<br><br>Adapted and extended based on previous studies of Biermann et al. (2018), Wilkowska et al. (2019) and Wilkowska (2015)	.93
**Negative Effects of Aging**<br><br>*"I think in older age,…*	1. ... I am less mobile and therefore socially more isolated due to health restrictions".<br>2. ... I am (strongly) limited in my activities.<br>3. ... I am dependent on others."<br>4. ... I no longer have so much fun in life."<br>5. … I take things more difficult than before."<br>6. ... I am a burden on my family."<br>7. ... my dignity is severely compromised."<br>8. ... I am in poor health."<br>9. ... my cognitive abilities are diminishing."<br>10. ... my privacy and right to intimacy are greatly reduced."<br>11. ... I have more to do with (medical) technical devices than with other people.<br>12. ... I become more and more fragile."<br>13. ... I can't do anything great in the rest of my life."	Based on analyses of Wurm et al., 2007<br><br>Adapted and extended based on previous studies of Biermann et al. (2018), Wilkowska et al. (2019) and Wilkowska (2015)	.95
**Perceived Benefits**	1. The measurement is reliable.<br>2. Enables quick access to health data.<br>3. Enables a trustworthy handling of data<br>4. Ensures that the state of health is checked regularly.<br>5. Gives the possibility of a quick reaction in case of emergency.<br>6. Enables control of the body functions from home.<br>7. Offers time saving in everyday life.<br>8. Increases your own mobility.<br>9. Enables rapid warning in case of critical vital values.<br>10. Relieves fellow human beings.<br>11. Offers a gain in safety.<br>12. Increases independence (autonomy).<br>13. Reduces dependence on others.<br>14. Creates relief in everyday life.	Based on literature review, e.g., Peek et al., 2014<br><br>Extended and adapted from previous studies, i.e., Wilkowska et al., 2019	.96
**Perceived Barriers**	1. There are doubts about the effectiveness of such a system.<br>2. It gives unauthorized persons access to personal health records.<br>3. Its use provokes stigmatization through the visibility of the disease.<br>4. The technology has a monitoring character.<br>5. The technology reminds of the disease.<br>6. The operation is difficult.<br>7. The system is too impersonal.<br>8. The proportion of technology is too high anyway.<br>9. Flooding by technology.<br>10. Perceived dependence on technology.<br>11. Lack of confidence in the functionality of the technology.<br>12. Lack of trust in the accuracy of the technology.<br>13. Human attention is replaced by technology.<br>14. Technology is an invasion of privacy.<br>15. Technology causes loneliness (isolation).	Based on literature review, e.g., Peek et al., 2014<br><br>Extended and adapted from previous studies, i.e., Wilkowska et al., 2019	.95
**Technology Acceptance**	1. I find the use of medical technology useful.<br>2. I do not want to use medical technology.<br>3. I can imagine the use of medical technology.	Based on Technology Acceptance Model (TAM) – Davis et al., 1989; 1993	.74
